# Sensitive Detection of Biomarker in Gingival Crevicular Fluid Based on Enhanced Electrochemiluminescence by Nanochannel-Confined Co_3_O_4_ Nanocatalyst

**DOI:** 10.3390/bios15010063

**Published:** 2025-01-19

**Authors:** Changfeng Zhu, Yujiao Zhao, Jiyang Liu

**Affiliations:** 1 Department of Stomatology, Beijing Hospital of Integrated Traditional Chinese and Western Medicine, Beijing 100039, China; 2024211001056@mails.zstu.edu.cn; 2 School of Chemistry and Chemical Engineering, Zhejiang Sci-Tech University, Hangzhou 310018, China; 2023211001071@mails.zstu.edu.cn

**Keywords:** electrochemiluminescence, immunosensor, nanochannel-confined, Co_3_O_4_, luminol

## Abstract

The sensitive detection of inflammatory biomarkers in gingival crevicular fluid (GCF) is highly desirable for the evaluation of periodontal disease. Luminol-based electrochemiluminescence (ECL) immunosensors offer a promising approach for the fast and convenient detection of biomarkers. However, luminol’s low ECL efficiency under neutral conditions remains a challenge. This study developed an immunosensor by engineering an immunorecognition interface on the outer surface of mesoporous silica nanochannel film (SNF) and confining a Co_3_O_4_ nanocatalyst within the SNF nanochannels to improve the luminol ECL efficiency. The SNF was grown on an indium tin oxide (ITO) electrode using the simple Stöber solution growth method. A Co_3_O_4_ nanocatalyst was successfully confined within the SNF nanochannels through in situ electrodeposition, confirmed by X-ray photoelectron spectroscopy (XPS) and electrochemical measurements. The confined Co_3_O_4_ demonstrated excellent electrocatalytic activity, effectively enhancing luminol and H_2_O_2_ oxidation and boosting the ECL signal under neutral conditions. Using interleukin-6 (IL-6) as a proof-of-concept demonstration, the epoxy functionalization of the SNF outer surface enabled the covalent immobilization of capture antibodies, forming a specific immunorecognition interface. IL-6 binding induced immunocomplex formation, which reduced the ECL signal and allowed for quantitative detection. The immunosensor showed a linear detection range for IL-6 from 1 fg mL^−1^ to 10 ng mL^−1^, with a limit of detection (LOD) of 0.64 fg mL^−1^. It also demonstrated good selectivity and anti-interference capabilities, enabling the successful detection of IL-6 in artificial GCF samples.

## 1. Introduction

Periodontal disease is a common chronic non-specific condition with a high prevalence, and it is one of the leading causes of tooth loss in middle-aged and elderly individuals [1]. However, the diagnosis and treatment of periodontal disease still face many challenges. Periodontal disease typically has a long course, with subtle early symptoms, and clinical diagnosis mainly relies on periodontal indices and X-ray digital subtraction techniques [2]. However, these methods have limitations, making it difficult to achieve an early assessment of periodontal disease and to assess its activity in a timely manner. In recent years, with the continuous advancement of molecular biology and biosensing technologies, research on biomarkers has gained widespread attention [3,4,5]. Gingival crevicular fluid (GCF) is the only bodily fluid that directly exudes from tissues, and its composition and concentration changes are closely related to the health status of periodontal tissues [6]. When inflammation occurs and progresses in the periodontium, the content of GCF increases, and its composition changes significantly, releasing a large number of inflammatory molecules. For example, inflammatory cells secrete cytokines such as interleukin-6 (IL-6) and IL-8, which are closely related to immune inflammation [7]. It has been proven that the levels of IL-6 in GCF and saliva are elevated in patients with periodontal disease and are correlated with the severity of the disease [8]. Additionally, GCF can be repeatedly collected and is non-invasive, minimizing patient discomfort. Therefore, the sensitive detection of inflammatory biomarkers in GCF is highly desirable for the quantitative evaluation of periodontal health.

Currently, common techniques for detecting inflammatory biomarkers in GCF include enzyme-linked immunosorbent assays (ELISAs), Western blotting, spectroscopy, and radioimmunoassays [9]. However, ELISA has limited sensitivity and Western blotting has low efficiency and requires larger sample volumes, while spectroscopy and radioimmunoassays are more restricted in their application due to the technical complexity and limitations in detectable targets [9]. Therefore, the development of novel, highly sensitive, and rapid detection technologies is crucial for the early diagnosis and precise treatment of periodontal disease. Electrochemiluminescence (ECL) detection combines the advantages of both electrochemistry and luminescence, offering unique benefits, and has garnered widespread attention [10,11,12]. Compared to traditional optical detection [13,14,15,16,17], ECL does not rely on external light sources, avoiding background interference and providing a higher signal-to-noise ratio [18,19,20,21]. Additionally, ECL offers excellent controllability, with the ability to precisely control the initiation and termination of the luminescent reaction by adjusting the potential, enabling highly selective and quantitative analysis [22]. ECL systems are typically simple in structure, easy to operate, and can be integrated with miniaturized devices, making them suitable for portable, real-time detection applications [23,24,25]. Moreover, ECL exhibits good versatility and scalability, as different electroactive materials and luminescent reagents can be selected to flexibly adjust the detection range and target species, making it widely applicable in clinical diagnostics, environmental monitoring, and food safety [26]. Thus, ECL detection is a highly promising technique for detecting inflammatory biomarkers in gingival crevicular fluid.

Luminol is a classic ECL emitter favored for its low cost, simple synthesis method, and ease of commercial supply, making it suitable for large-scale applications [27]. Luminol-based ECL immunosensors hold promise for the fast and convenient detection of biomarkers. However, luminol has a relatively low quantum yield, and its luminescence intensity under neutral conditions is much lower than that under alkaline conditions [28]. Recent studies have shown that combining luminol with nanomaterials can enhance its ECL intensity under neutral conditions, providing an effective approach for developing highly sensitive ECL analytical platforms [29]. Nanomaterials possess a high surface area, excellent catalytic activity, and good optical and electrical properties, which can significantly increase the ECL efficiency of luminol [30,31,32]. For instance, nanomaterials such as nanocatalysts or transition metal nanomaterials can catalyze the oxidation reaction of luminol, increasing the reaction rate and enhancing the ECL signal [33]. On the other hand, nanomaterials can catalyze the generation of reactive oxygen species [34,35,36]. However, the stability of nanomaterials on electrodes remains challenging.

Confining nanomaterials within porous materials is an effective strategy for enhancing their stability [37,38,39]. The confinement effect of porous materials physically encapsulates or embeds nanomaterials, reducing aggregation and thus improving their stability [40]. In recent years, the confinement of nanomaterials within silica nanochannel film (SNF) has garnered significant attentions [41,42,43]. SNF is a functional film based on silica materials, featuring precisely controlled mesoporous nanostructures (typically 2–3 nm in pore size) and broad applications in catalysis, separation, sensing, and energy storage [44,45,46,47,48]. Their unique physicochemical properties offer distinct advantages, including highly ordered nanostructures, high surface area, chemical stability, optical transparency, and biocompatibility [49,50,51]. SNF is typically synthesized using a templating method, enabling controllable pore sizes (2–20 nm) with uniform pore distribution. As a result, SNF can selectively filter and transport specific molecules [52,53]. For example, the size-exclusion properties of SNF nanochannels allow for the removal of macromolecules and particulates from complex samples [54,55,56], while the negatively charged surface created by silanol ionization can repel common negatively charged electroactive substances such as uric acid (UA) and ascorbic acid (AA) [57]. The nanochannels also significantly increase the surface area, providing more active sites for chemical reactions, and the external surface can be further functionalized biologically [58,59,60,61]. Thus, utilizing SNF to confine functional nanomaterials with luminol as an enhanced ECL offers a promising approach for developing highly sensitive biosensors for biomarker detection.

In this work, mesoporous silica nanochannel film (SNF) with dual functional domains was used to modify the cheap electrode, enabling the construction of an immunosensor interface and the stable confinement of nanocatalysts. An SNF-modified electrode was prepared using a straightforward method, with an immunorecognition interface constructed on its outer surface and stable Co_3_O_4_ nanocatalysts confined within the nanochannels to enhance the luminol ECL signal under near-neutral conditions. Using interleukin-6 (IL-6) as a proof-of-concept demonstration, the constructed immunosensor successfully displayed its effectiveness as a biomarker. Upon the presence of IL-6, the specific immunorecognition increased the interfacial resistance, and the formation of the immunocomplex hindered luminol diffusion, subsequently reducing the ECL signal. Based on this mechanism, the sensitive detection of IL-6 was successfully achieved. The fabricated immunosensor demonstrated good selectivity and anti-interference properties, making it suitable for IL-6 detection in complex biological matrices.

## 2. Materials and Methods

### 2.1. Chemicals and Materials

Cetyltrimethylammonium bromide (CTAB) and tetraethyl orthosilicate (TEOS) were purchased from Sigma-Aldrich (Shanghai, China). Luminol, hydrogen peroxide (H_2_O_2_), disodium hydrogen phosphate dodecahydrate (Na_2_HPO_4_·12H_2_O), sodium dihydrogen phosphate dihydrate (NaH_2_PO_4_·2H_2_O), potassium ferricyanide (K_3_[Fe(CN)_6_]), potassium ferrocyanide (K_4_[Fe(CN)_6_]), ruthenium(III) hexammine chloride (Ru(NH_3_)_6_Cl_3_), ferrocenemethanol (FcMeOH), potassium chloride (KCl), potassium hydrogen phthalate (KHP), cobalt(II) sulfate heptahydrate (CoSO_4_·7H_2_O), (3-glycidyloxypropyl)trimethoxysilane (GPTMS), sodium hydroxide (NaOH), and bovine serum albumin (BSA) were obtained from Aladdin Reagent Co., Ltd. (Shanghai, China). Interleukin-6 antigen (IL-6) and IL-6 monoclonal antibody (Ab) were purchased from Keyuezhongkai Biotech Co., Ltd. (Beijing, China). Artificial gingival crevicular fluid (GCF) was purchased from Chemazone Inc. (Nashville, TN, USA). All solutions used in the experiments were prepared with ultrapure water (18.2 MΩ·cm) obtained from a Mill-Q system (Millipore, IL, USA). Indium tin oxide (ITO) conductive glass was purchased from Zhuhai Kaiyue Electronic Components Co., Ltd. (Zhuhai, China). Prior to use, ITO glass was immersed in an aqueous NaOH solution (1 M) for 12 h to remove organic residues. It was then sonicated in acetone and ethanol for 30 min each, followed by sonication in ultrapure water for 10 min. After drying with nitrogen gas, the ITO glass was then cut into electrode pieces of 0.5 cm × 5 cm using a glass cutter with an active area of 0.5 cm × 1 cm defined by insulating tape.

### 2.2. Measurements and Instrumentations

The morphology and thickness of SNF were characterized using transmission electron microscopy (TEM) and scanning electron microscopy (SEM). To prepare the TEM sample, the SNF layer was carefully scraped from the electrode with a scalpel and then dispersed in anhydrous ethanol followed by ultrasonic treatment. The resulting dispersion was drop-cast onto a copper grid and air-dried. Then, the sample was observed with the TEM instrument (JEOL JEM-2100Plus, JEOL Ltd., Tokyo, Japan). For SEM analysis, the sample required gold coating prior to observation. To examine the cross-section of the sample, a glass cutter was used to gently score the back of the SNF/ITO, then a fresh cross-section was created by breaking it, followed by gold coating and SEM observation (SU8010, Hitachi, Tokyo, Japan). X-ray photoelectron spectroscopy (XPS) was performed using a PHI5300 spectrometer (PE Ltd., Boston, MA, USA), with a Mg Kα radiation source (250 W, 14 kV). Cyclic voltammetry (CV) and electrochemical impedance spectroscopy (EIS) measurements were conducted on a PGSTAT302N electrochemical workstation (Autolab, Metrohm, Heilissau, Switzerland), using a conventional three-electrode system. Specifically, ITO or modified ITO was used as the working electrode, a platinum wire or platinum foil was used as the counter electrode, and an Ag/AgCl electrode (saturated KCl) was used as the reference electrode. Parameters for differential pulse voltammetry (DPV) tests included a step potential of 0.005 V, a pulse amplitude of 0.025 V, a pulse time of 0.05 s, and an interval time of 0.2 s. Electrochemiluminescence (ECL) tests were conducted on an MPI-E II instrument (Remex Analytical Instrument Co., Ltd., Xi’an, China).

### 2.3. Preparation of the Immunosensor

Using the Stöber solution growth method, SNF was grown on the ITO surface [62,63]. Specifically, 0.160 g of CTAB was dissolved in a mixed solution of 70 mL ultrapure water and 30 mL anhydrous ethanol. Under stirring, 100 μL of 10% ammonia solution and 80 μL of TEOS were rapidly added, and the solution was stirred for an additional 5 min until it was bubble-free, obtaining the precursor solution. A clean ITO glass was immersed in the precursor solution and reacted in a 60 °C water bath for 24 h. Afterward, the electrode was removed, rinsed with ultrapure water, dried with nitrogen, and aged overnight at 100 °C, resulting in an electrode containing surfactant micelles (SMs) in nanochannels (SM@SNF/ITO).

To achieve the covalent binding of recognition antibodies, the outer surface of the SNF was functionalized with silanes containing epoxy groups, introducing the epoxy groups. Subsequently, the covalent immobilization of antibodies was achieved through the ring-opening reaction between the epoxy groups and the amine groups on the antibodies. Specifically, the SM@SNF/ITO electrode was immersed in 50 mL of 2.26 mM GPTMS ethanol solution and reacted at 25 °C for 1 h to achieve epoxy group modification. After the reaction, the electrode was rinsed with ultrapure water to obtain an electrode with epoxy-modified outer surface SNF (SM@O-SNF/ITO). The SM@O-SNF/ITO was then immersed in an HCl-ethanol solution to remove micelles within the nanochannels, producing an electrode with open nanochannels (O-SNF/ITO).

Subsequently, in situ deposition of Co_3_O_4_ nanocatalyst was achieved in the nanochannels via electrodeposition. Specifically, the O-SNF/ITO was immersed in 10 mL of 0.2 M CoSO_4_ solution, and electrodeposition was conducted at 1.5 V for 15 s, resulting in an electrode with nanochannel-confined Co_3_O_4_ (Co_3_O_4_@O-SNF/ITO).

To prepare the immunorecognition interface, the Co_3_O_4_@O-SNF/ITO electrode was immersed in an amino-modified IL-6 antibody solution and incubated at 4 °C for 1 h. The electrode was then thoroughly washed with 0.01 M PBS (pH 7.4), and the antibody covalently immobilized electrode was denoted as Ab/Co_3_O_4_@O-SNF/ITO. Next, the Ab/Co_3_O_4_@O-SNF/ITO was immersed in a 1% BSA solution (0.01 M PBS, pH 7.4) and incubated at room temperature for 15 min to block non-specific binding sites, yielding the immunosensor (BSA/Ab/Co_3_O_4_@O-SNF/ITO). The immunosensor was stored at 4 °C until use.

### 2.4. ECL Detection of IL-6

The immunosensor was incubated with various concentrations of IL-6 for 60 min. The ECL signal on the electrode after IL-6 binding was measured in an electrochemical support solution containing 100 μM H_2_O_2_ and 100 μM luminol in PBS (0.01 M, pH = 7.4). To investigate the reproducibility of the electrode detection, five electrodes were prepared in parallel and used to measure 10 ng mL^−1^ of IL-6. The relative standard deviation (RSD) was measured. The ECL process was triggered by a continuous CV program, with a potential scan range of 0–0.8 V and a scan rate of 0.1 V/s. The photomultiplier tube (PMT) voltage was set to 750 V.

### 2.5. Detection of IL-6 in GCF

The concentration of IL-6 in artificial GCF was determined using the standard addition method. After adding standard IL-6 solutions of varying concentrations to GCF, the samples were diluted 50-fold with PBS, and the IL-6 content was measured to evaluate the recovery and relative standard deviation (RSD) of the assay.

## 3. Results and Discussion

### 3.1. Strategy for Immunosensor Construction and ECL Sensing

**Figure 1:**
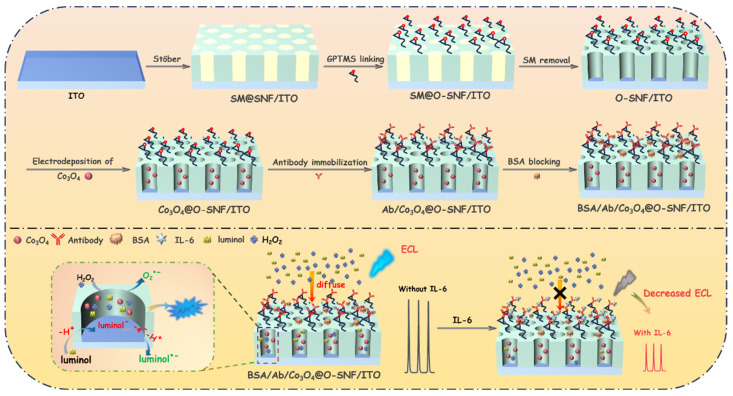
Schematic illustration for immunosensor construction and ECL detection of IL-6 through integrating both a specific recognition interface on the outer surface of SNF and Co_3_O_4_ nanocatalyst confined in SNF nanochannels.

As shown in Figure 1, this work involved the modification of both the outer surface and nanochannels of SNF to fabricate an immunosensor for highly sensitive ECL detection of IL-6. To reduce costs, inexpensive and readily available indium tin oxide (ITO) conductive glass was used as the base electrode, and the Stöber solution growth method was applied to grow SNF on its surface. This Stöber method utilized cationic surfactant micelles (SMs) as a template, where the SM-templated siloxane self-assembly enabled the efficient, one-step synthesis of SNF-modified electrodes [62,63]. The resulting SNF-modified electrode contained two functional regions. First, the outer surface was functionalized with reactive epoxy groups, facilitating the covalent immobilization of the recognition antibody (Ab). After antibody immobilization, non-specific sites were blocked with BSA to create a specific immunorecognition interface. Second, the nanochannels served as confined spaces for the in situ growth of the cobalt oxide (Co_3_O_4_) nanocatalyst. To ensure that epoxy group derivatization occurred on the outer surface of the SNF, derivatization was performed on the electrodes containing SM within the nanochannels. The SM blocked the nanochannels, allowing epoxy group derivatization to occur only on the outer surface of the SNF. The SM template was then removed by immersing the electrode in a 0.1 M HCl-ethanol solution with stirring, resulting in an SNF-modified electrode with open nanochannel arrays and epoxy-functionalized surfaces. Thus, this immunosensor design integrated both a specific recognition interface and a nanocatalytic region. Using luminol as the ECL emitter, hydrogen peroxide (H_2_O_2_) as a co-reactant, and Co_3_O_4_ confined within the nanochannels as a co-reaction accelerator, the sensor possessed an enhanced ECL signal. When the immunorecognition interface selectively captured IL-6, the formation of an immunocomplex hindered the diffusion of luminol and H_2_O_2_, reducing the ECL signal on the electrode. This mechanism enabled the sensitive detection of IL-6.

### 3.2. Characterization of SNF-Modified Electrodes

**Figure 2:**
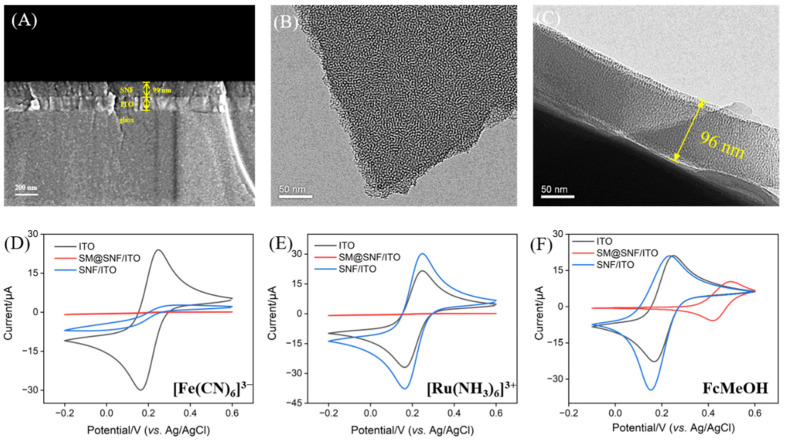
(**A**) SEM image of the cross-section of SNF/ITO electrode. (**B**) Top-view TEM image of SNF. (**C**) Cross-sectional TEM image of SNF. (**C**–**E**) CV curves obtained on ITO, SM@SNF/ITO, and SNF/ITO electrodes in 0.05 M KHP (pH 4) containing 0.5 mM of K_3_Fe(CN)_6_ (**D**), Ru(NH_3_)_6_Cl_3_, (**E**) or FcMeOH (**F**). The scan rate was 50 mV/s.

Figure 2A shows a cross-sectional SEM image of the SNF-modified electrode (SNF/ITO). As shown, the electrode exhibits a three-layer structure, with the SNF layer on top, followed by the ITO conductive layer and glass substrate of the conduction ITO glass. In addition, the SNF layer has a smooth surface, with a measured film thickness of ~99 nm. Figure 2B displays a top-view TEM image of the SNF, in which each bright pot represents a nanochannel. It is revealed that the SNF layer is an intact, crack-free layer with worm-like nanochannels. The cross-sectional TEM image of the SNF (Figure 2C) indicates a thickness of approximately 96 nm, consistent with the SEM measurement.

The completeness and charge-selective permeability of the SNF were evaluated by measuring the signals of electrochemical probes on different electrodes using cyclic voltammetry (CV) (Figure 2D–F). Three standard redox probes were selected including anionic Fe(CN)_6_^3−^, cationic Ru(NH_3_)_6_^3+^, and neutral ferrocene methanol (FcMeOH). As shown in Figure 2D,E, the bare ITO electrode exhibited clear redox peaks in solutions of Fe(CN)_6_^3−^ and Ru(NH_3_)_6_^3+^. However, with SNF added onto the electrode and surfactant micelles (SMs) retained within the channels (SM@SNF/ITO), ion transport was blocked by the presence of SMs within the channels, resulting in an almost complete absence of redox signals for both probes. This indicated the intactness of the SNF. Following the removal of the SMs, the open-nanochannel-modified electrode (SNF/ITO) showed a significant suppression of the Faradaic current for the anionic probe and an enhancement for the cationic probe compared to ITO alone, indicating the selective repulsion of anions and the attraction of cations. This behavior was attributed to the negative surface charge on the SNF, originating from the ionization of silanol groups, which repelled anionic species and attracted cationic ones. Figure 2F displays the CV curves of the FcMeOH solution for various electrodes. With the SMs retained, FcMeOH reached the electrode surface via SM-facilitated enrichment, producing a redox signal. However, this process consumed additional energy, causing the redox peak potential to shift positively. Furthermore, as the oxidation product of FcMeOH carried a positive charge, it was enriched by the negatively charged SNF channels, resulting in a reduction peak current higher than the oxidation peak current for SNF/ITO. These results confirmed the successful growth of the SNF on the ITO electrode, which displayed structural integrity and ion-selective permeability.

### 3.3. Characterization of Co_3_O_4_-Confined Electrode

**Figure 3:**
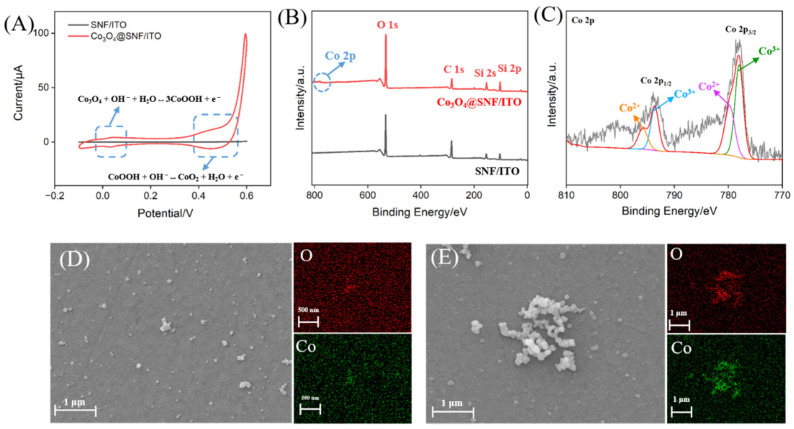
(**A**) CV curves obtained on SNF/ITO or Co_3_O_4_@SNF/ITO in 1 M NaOH. The scanning rate was 100 mV/s, and the scanning potential ranged from –0.1 V to 0.6 V. (**B**) XPS spectra obtained on the fabricated SNF/ITO or Co_3_O_4_@SNF/ITO electrode. (**C**) High-resolution Co 2p spectrum obtained on Co_3_O_4_@SNF/ITO electrode. (**D**) SEM image (**left** image) of Co_3_O_4_@SNF/ITO electrode after removal of SNF through immersion into a 0.5 M NaOH solution for 3 min and the corresponding O (**right** and **above** image) and Co (**right** and **bottom** image) element mapping image. (**E**) Top-view SEM image (**left** image) of Co_3_O_4_@/ITO electrode and the corresponding O (**right** and **above** image) and Co (**right** and **bottom** image) element mapping image.

To confirm the successful confinement of Co_3_O_4_ within the SNF nanochannels, the Co_3_O_4_@SNF/ITO electrode was characterized. Figure 3A shows the CV curves obtained on the SNF/ITO and Co_3_O_4_@SNF/ITO electrodes in 1 M NaOH solution before and after Co_3_O_4_ confinement. It was observed that the Co_3_O_4_@SNF/ITO electrode displayed two characteristic redox peaks associated with cobalt. During CV scanning, Co_3_O_4_ underwent oxidation to form CoOOH, which was further oxidized to CoO_2_. X-ray photoelectron spectroscopy (XPS) was used to analyze the elemental composition of the Co_3_O_4_@SNF/ITO electrode. Figure 3B shows the XPS survey spectrum for the Co_3_O_4_@SNF/ITO electrode, where a Co 2p peak appeared near 779 eV, confirming Co_3_O_4_ confinement compared to the SNF/ITO electrode. Figure 3C presents the high-resolution Co 2p spectrum, showing a peak at 779.4 eV as a characteristics of the Co_3_O_4_ material. Additionally, a 14.6 eV energy band gap between the Co 2p_1/2_ and Co 2p_3/2_ peaks was observed, which was another characteristic feature of Co_3_O_4_.

SEM was used to verify the deposition of Co_3_O_4_ within the nanochannels. After dissolving the SNF on the Co_3_O_4_@SNF/ITO surface with NaOH, SEM characterization was performed, as shown in Figure 3D. A large number of nanoparticles appeared on the ITO electrode surface, and the signals of Co and O in the element mapping confirmed that the material was Co_3_O_4_. In comparison, the SEM image of the Co_3_O_4_/ITO electrode, which was directly electrodeposited on the ITO electrode (Figure 3E), revealed nanoparticles of varying sizes and larger particles. Element mapping confirmed that the structure was composed of Co_3_O_4_ nanoparticles. Compared to the Co_3_O_4_ on the electrode surface after SNF dissolution, the Co_3_O_4_ on the Co_3_O_4_/ITO electrode was larger in size and tended to aggregate. It was speculated that during the direct electrodeposition of Co_3_O_4_ on the ITO surface, aggregation occurred due to the absence of protective agents or confinement spaces. In contrast, the 2D rigid structure of the nanochannel array could limit the growth of the nanomaterials, stabilizing them within the nanochannels. After the SNF on the surface was dissolved, Co_3_O_4_ aggregated in the absence of the protective nanochannels. The above results indirectly confirmed that the electrodeposited Co_3_O_4_ was successfully confined within the nanochannels.

### 3.4. Enhanced ECL Signal of the Luminol-H_2_O_2_ System by Confined Co_3_O_4_

**Figure 4:**
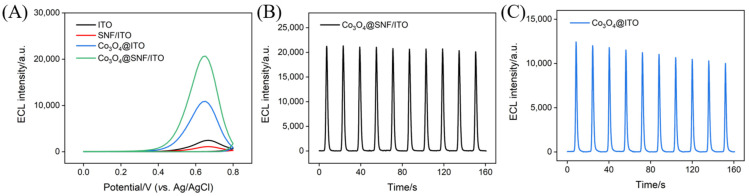
(**A**) ECL curves obtained from different electrodes in PBS (0.01 M, pH 7.4) containing luminol (100 μM) and H_2_O_2_ (100 μM). (**B**) ECL intensity obtained on Co_3_O_4_@SNF/ITO or (**C**) Co_3_O_4_/ITO from continuously scans in PBS (0.01 M, pH 7.4) containing luminol (100 μM) and H_2_O_2_ (100 μM). The PMT voltage was set to 750 V. The scanning rate was 100 mV/s, and the scanning potential range was 0 V~0.8 V.

Figure 4A presents the ECL signals measured from the ITO, SNF/ITO, Co_3_O4@ITO, and Co_3_O_4_@SNF/ITO electrodes in PBS solution (0.01 M, pH 7.4) containing luminol and the co-reactant H_2_O_2_. Compared to the ITO electrode, the ECL signal recorded on the SNF/ITO electrode was significantly reduced, which was likely attributed to the repulsive interaction between the negatively charged SNF surface and the active luminol anions. In contrast, the presence of Co_3_O_4_ significantly enhanced the ECL signals for both the Co_3_O_4_@ITO and Co_3_O_4_@SNF/ITO electrodes, demonstrating the catalytic role of Co_3_O_4_ in boosting the ECL signal. Moreover, the Co_3_O_4_ confined in the SNF exhibited a more pronounced enhancement effect on the ECL signal. Specifically, the ECL signal recorded on the Co_3_O_4_@SNF/ITO electrode was twice as high as that on the Co_3_O_4_@ITO electrode, where Co_3_O_4_ was directly grown on the ITO surface. This enhancement was likely due to the vertically ordered nanochannel array structure, which effectively suppressed the aggregation of Co_3_O_4_ nanomaterials and facilitated the formation of more catalytically active Co_3_O_4_.

Additionally, the stability of Co_3_O_4_ confined within the SNF or directly grown on ITO was investigated. As shown in Figure 4B, the Co_3_O_4_@SNF/ITO electrode exhibited a relative standard deviation (RSD) of only 1.8% for ECL intensity during continuous CV scans, demonstrating the stability of confined Co_3_O_4_ within the nanochannels. In contrast, the RSD of the ECL intensity for the Co_3_O_4_ deposited on the ITO electrode (Co_3_O_4_/ITO) was 7.2% under the same conditions (Figure 4C). Therefore, confining Co_3_O_4_ within the nanochannel array of the SNF not only significantly enhanced the ECL intensity of the electrode but also ensured the high stability of Co_3_O_4_. On one hand, the presence of nanochannels effectively limited the growth of the nanomaterials, resulting in smaller-sized Co_3_O_4_ with a larger specific surface area. On the other hand, the stable nanochannels on the electrode surface confined the generated Co_3_O_4_, reducing its detachment and thereby improving stability. In contrast, Co_3_O_4_ directly deposited on the bare ITO electrode tended to form larger-sized or aggregated nanomaterials, and Co_3_O_4_ was more likely to detach from the electrode surface during measurements, leading to a lower signal and decreased stability.

### 3.5. Mechanism of Co_3_O_4_-Enhanced ECL Signal

**Figure 5:**
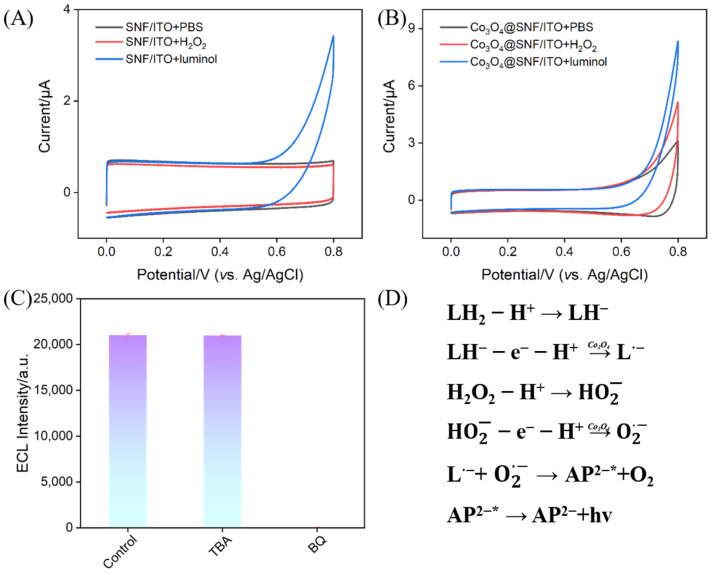
CV curves obtained on SNF/ITO (**A**) and Co_3_O_4_@SNF/ITO (**B)** electrodes in the electrolyte (PBS, 0.01 M, pH 7.4) or electrolyte containing luminol (100 μM) or H_2_O_2_ (100 μM). (**C**) ECL intensity obtained at the Co_3_O_4_@SNF/ITO electrode in PBS (0.01 M, pH 7.4) containing luminol (100 μM) and H_2_O_2_ (100 μM) in the presence of TBA (100 μg mL^−^^1^) or BQ (100 μM). (**D**) Illustration of possible ECL mechanism for luminol-H_2_O_2_ system enhanced by Co_3_O_4_ nanomaterials.

To further investigate the catalytic role of Co_3_O_4_ in enhancing the ECL signal of the luminol-H_2_O_2_ system, CV tests were conducted on confined and unconfined Co_3_O_4_ nanomaterial-modified electrodes in the electrolyte (PBS, 0.01 M, pH 7.4) or electrolyte containing luminol or H_2_O_2_. As shown in Figure 5A,B, the SNF/ITO electrode exhibited no faradaic current response in PBS. However, the Co_3_O_4_@SNF/ITO electrode showed a current response of 3.78 μA at 0.8 V, indicating that the Co_3_O_4_ nanomaterials promoted the oxygen evolution reaction (OER). Upon the addition of luminol, the current signal increased to 8.42 μA, confirming that the Co_3_O_4_ nanomaterials catalyzed the electrochemical oxidation of luminol. Similarly, in the presence of H_2_O_2_, the Co_3_O_4_@SNF/ITO electrode exhibited a current response of 5.64 μA at 0.8 V, of which 1.86 μA was attributed to the electrocatalytic oxidation of H_2_O_2_ after subtracting the OER contribution (3.78 μA). Thus, the confined Co_3_O_4_ nanomaterials possess electrocatalytic oxidation capabilities for both luminol and H_2_O_2_, leading to the generation of more reactive species and, consequently, enhancing the ECL signal.

The catalytic oxidation of H_2_O_2_ by Co_3_O_4_ nanomaterials generated reactive oxygen species (ROS), which amplified the luminol ECL signal. To identify the types of radicals involved, radical scavenging experiments were conducted using hydroxyl radical scavenger tert-butanol (TBA) and superoxide radical scavenger benzoquinone (BQ). As shown in Figure 5C, the ECL signal remained unchanged after adding TBA, indicating that ·OH was not the main radical in the reaction. However, the ECL signal dropped to nearly zero upon the addition of BQ, confirming that the ROS produced by Co_3_O_4_ during the catalytic oxidation of H_2_O_2_ was a superoxide radical (O_2_^−^·).

Based on the experimental results, the proposed mechanism for the ECL signal enhancement by confined Co_3_O_4_ nanomaterials in the luminol-H_2_O_2_ system is illustrated in Figure 5D. In a weakly alkaline solution, luminol (LH_2_) loses a proton to form luminol^−^ (LH^−^). Under the catalysis of confined Co_3_O_4_ nanomaterials, LH^−^ undergoes electron and proton loss to generate the luminol radical (L•^−^). Simultaneously, H_2_O_2_ is catalyzed by Co_3_O_4_ to produce O_2_^−^·. The L•^−^ reacts with O_2_^−^· on the electrode surface to form an excited state (AP^2^^−^*), which returns to the ground state with light emission, generating the ECL signal.

### 3.6. Characterization of Immunosensor Construction and Feasibility for IL-6 Detection

**Figure 6:**
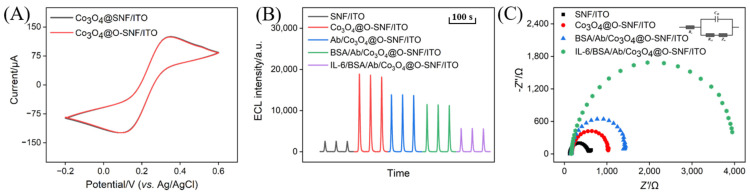
(**A**) CV curves obtained on Co_3_O_4_@O-SNF/ITO or Co_3_O_4_@O-SNF/ITO electrodes in 0.1 M KCl containing 2.5 mM [Fe(CN)_6_]^3−/4−^. (**B**) ECL responses obtained on different electrodes in PBS (0.01 M, pH 7.4)) with H_2_O_2_ (100 μM) and luminol (100 μM). (**C**) EIS plots obtained on different electrodes in 0.1 M KCl containing 2.5 mM [Fe(CN)_6_]^3−/4−^.

Figure 6A shows the cyclic voltammetry (CV) curve obtained on Co_3_O_4_@O-SNF/ITO in Fe(CN)_6_^3−^/^4−^ solution, which was derived with epoxy groups before the confinement of Co_3_O_4_ in the SNF nanochannels. For comparison, the Co_3_O_4_@SNF/ITO electrode without epoxy functionalization was also investigated. As observed, both electrodes exhibited consistent CV signals. This was attributed to the selective epoxy functionalization conducted on the SNF external surface in the presence of SMs during the immunosensor construction process. The SMs effectively blocked the SNF nanochannels, ensuring that the functionalization occurred only on the external surface, thereby avoiding any adverse effects on the nanochannel structure.

The feasibility of immunosensor construction was further investigated by examining the ECL signals measured on the electrodes modified stepwise. As shown in Figure 6B, confining Co_3_O_4_ within the nanochannels significantly enhanced the ECL signal due to the catalytic activity of Co_3_O_4_. However, when Ab was covalently immobilized on the external surface of the SNF and the non-specific sites were blocked with BSA, the ECL signal decreased. This reduction was attributed to the protein characteristics of Ab and BSA, which increased the interfacial resistance of the electrode. When IL-6 specifically bound to Ab, forming an immunocomplex, the ECL signal further decreased significantly. This can be explained by two factors. On the one hand, the immunocomplex increased the interfacial resistance of the electrode. On the other hand, the immunocomplex decreased the diffusion of luminol and H_2_O_2_ to the electrode surface. These dual effects contributed to the reduction in the ECL signal.

The above process was further characterized by electrochemical impedance spectroscopy (EIS), as shown in Figure 6C. The charge transfer resistance (R_ct_) of the SNF/ITO electrode was 424 Ω, which increased to 733 Ω after confining Co_3_O_4_ within the nanochannels (Co_3_O_4_@SNF/ITO). After constructing the immunorecognition interface and blocking non-specific sites with BSA, the Rct of the BSA/Ab/Co_3_O_4_@SNF/ITO electrode further increased to 1170 Ω. This increase was also due to the higher interfacial resistance caused by Ab and BSA. Finally, when IL-6 specifically bound to the recognition antibody, the Rct increased significantly to 3540 Ω. These results demonstrate the feasibility of the immunosensor construction and the potential for IL-6 detection.

### 3.7. Optimization of Immunosensor Construction and IL-6 Detection Conditions

**Figure 7:**
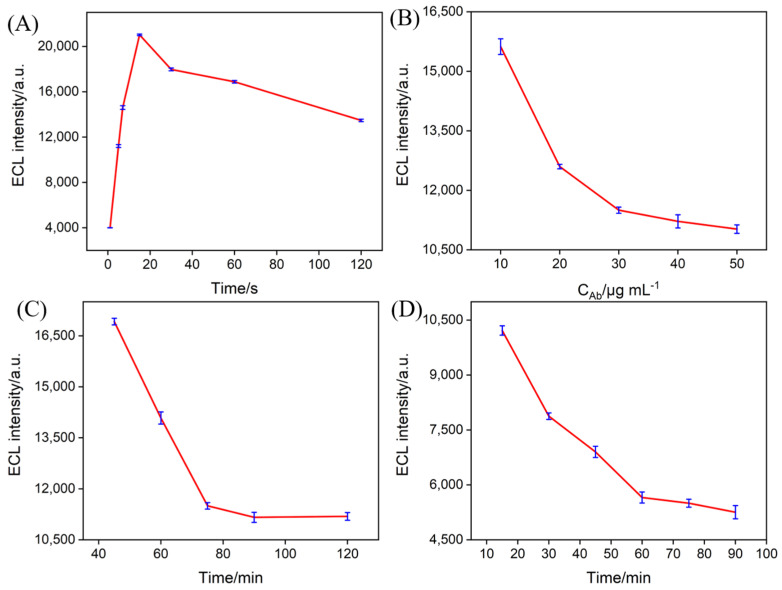
The effect of deposition time of Co_3_O_4_ (**A**), antibody concentration (**B**), incubation time for antibody immobilization (**C**), and IL-6 incubation time (**D**) on the ECL signals of the fabricated immunosensors.

To achieve optimal detection performance, key parameters of the constructed immunosensor were optimized, including the Co_3_O_4_ deposition time, antibody incubation concentration and incubation time for immobilization, and IL-6 binding time. Figure 7A shows the ECL signals of electrodes prepared with different Co_3_O_4_ deposition times. As illustrated, the ECL intensity of the electrode initially increased with longer deposition times but then began to decrease. This trend can be attributed to the increasing amount of deposited Co_3_O_4_ with extended deposition time, which enhanced the catalytic activity. However, excessive deposition may lead to the blockage of nanochannels, hindering mass transfer, ultimately reducing the ECL signal. Thus, the optimal deposition time was determined to be 15 s. Considering both the experimental cost and time efficiency, the antibody concentration and its incubation time were optimized in the fabrication of the immuonrecognition interface. As shown in Figure 7B,C, the ECL signal gradually decreased with increasing antibody concentration and incubation time. When the antibody concentration reached 30 μg mL^−^^1^ and the incubation time was 75 min, the ECL signal stabilized. Further increases in the antibody concentration or incubation time had minimal effects on the ECL signals, indicating that antibody immobilization had reached saturation under these conditions. Thus, an antibody concentration of 30 μg mL^−^^1^ and an incubation time of 75 min were selected for the subsequent studies. To optimize the binding time of the IL-6, the ECL signal was measured at various binding times. As shown in Figure 7D, the ECL signal reached its maximum when the binding time was 60 min. This was chosen for further investigation.

### 3.8. Electrochemiluminescence Detection of IL-6

**Figure 8:**
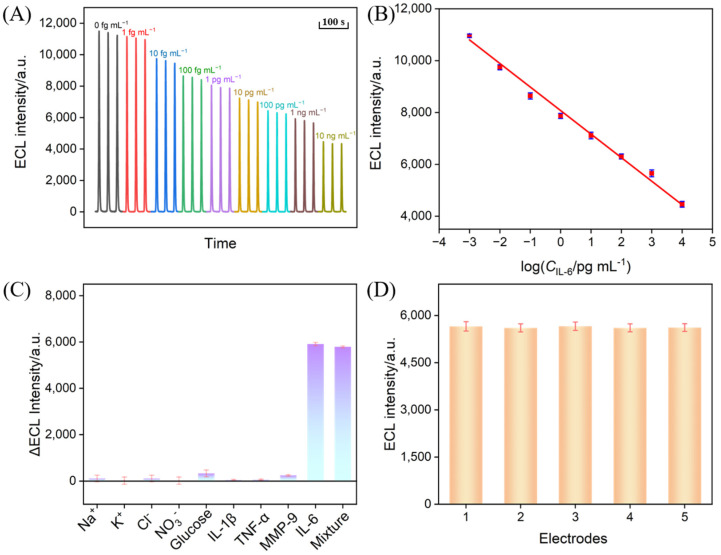
(**A**) ECL responses of the fabricated immunosensor in presence of various concentrations of IL-6 in PBS (0.01 M, pH 7.4) containing luminol (100 μM) and H_2_O_2_ (100 μM). (**B**) The corresponding calibration curves between ECL intensity and the logarithmic concentration of IL-6. (**C**) Reproducibility of five immunosensors fabricated in parallel for IL-6 detection (10 ng mL^−^^1^). (**D**) The selectivity and anti-interference of ECL immunosensor for the detection of IL-6. The concentration of Na^+^, Cl^−^ was 1 μM, the concentration of K^+^, NO^3−^ was 100 nM, the concentration of glucose was 10 μM, and the concentration of IL-1β, MMP-9, TNF-α was 10 ng mL^−^^1^.

Under the optimized conditions, the constructed immunosensor was incubated with various concentrations of IL-6, and the ECL signals of the electrode were measured in the luminol-H_2_O_2_ system (Figure 8A). It was observed that the ECL signal decreased with increasing IL-6 concentration. This phenomenon was attributed to the formation of immunocomplexes, which increased the interfacial resistance of the electrode and reduced the diffusion of luminol and H_2_O_2_. In the concentration range of 1 fg mL^−^^1^ to 10 ng mL^−^^1^, the ECL intensity (I_ECL_) exhibited a linear relationship with the logarithm of the IL-6 concentration (logC). The limit of detection (LOD) was calculated to be 0.64 fg mL^−^^1^ based on a signal-to-noise ratio of 3 (S/N = 3). The analytical performance of the different modified electrodes for the detection of IL-6 by ECL or electrochemical (EC) methods is summarized in Table S1 (in Supporting Information-SI) [64,65,66,67,68,69,70]. The LOD obtained on the fabricated immunosensor was lower than that obtained on an EC aptasensor based on a gold nanoparticle/polypyrrole-modified screen-printed gold electrode (AuNPs/PPyNPs/SPGE) [64], an EC immunosensor using a gold nanoparticle/reduced graphene oxide-modified Au electrode (AuNPs/rGO/Au) [65], an electrochemically reduced graphene oxide/gold palladium nanoparticle-modified heated carbon paste electrode (ErGO/AuPdNPs/HCPE) [66], a sandwich-type EC immunoassay based on ferrocene-porous polyelectrolyte nanoparticle-antibody 2/IL-6/antibody 1 on a graphene oxide modified glassy carbon electrode (FC-PPN-Ab_2_/IL-6/Ab_1_/GO/GCE) [67], an ECL immunosensor based on a reduced graphene oxide/Fe_3_O_4_/polydimethyl diallyl ammonium chloride/cadmium selenide nanoparticle-modified glassy carbon electrode (rGO/Fe_3_O_4_/PDDA/CdSe/GCE) [68], and a graphene oxide/polyaniline/cadmium selenide nanoparticle-modified glassy carbon electrode (BSA/Ab/GO/PANi/CdSe/GCE) [69]. The LOD was higher than that obtained using a sandwich-type ECL immunoassay using horseradish peroxidase-labeled antibody on acid phosphatase and octahedral anatase mesocrystals and a carboxyl-terminated ionic liquid-tris(2,2‘-bipyridyl)ruthenium(II) chloride-loaded TiO_2_ (anatase)mesocage-modified glassy carbon electrode (Ab_2_-HRP/ACP/OAMs/IL-6/BSA/Ab_1_/CTIL/Ru(bpy)_3_^2+^@AMCs/GCE) [70]. Thus, the fabricated immunosensor has advantages of simple construction and high detection performance.

### 3.9. Reproducibility, Selectivity, and Anti-Interference Capability of the ECL Sensor

To evaluate the performance of the sensor, its reproducibility, selectivity, and anti-interference capability were assessed. Five immunosensors fabricated in parallel were incubated with IL-6 solutions of the same concentration followed by the measurement of the ECL signal. The results demonstrated an RSD of 0.5% of the ECL response, indicating the good reproducibility of the fabricated immunosensors (Figure 8C).

Pro-inflammatory cytokines such as interleukin-1β (IL-1β) and tumor necrosis factor-α (TNF-α) are potential biomarkers associated with periodontal inflammation. In addition, periodontal inflammation can produce various cytokines, including interferon-γ (IFN-γ) and matrix metalloproteinases (MMPs). Thus, IL-1β, TNF-α, and MMP-9, as well as common inorganic salts such as sodium, potassium, and glucose found in the oral cavity, are selected as potential interferents to examine the selectivity and resistance to interference of the fabricated immunosensor. The results showed that significant changes in the ECL signal were observed only when the target analyte IL-6 or IL-6-containing mixtures were introduced (Figure 8D). The presence of other interfering substances had negligible effects on the signal. These results confirmed that the immunosensor possessed excellent selectivity and displayed a good anti-interference performance.

### 3.10. Real Sample Analysis

The performance of the prepared immunosensor for detecting IL-6 in real samples was evaluated using the standard addition method. Artificial GCF containing different concentrations of IL-6 were incubated with the constructed immunosensor, and the ECL signals of the electrodes were measured. As shown in Table 1, the recovery rates ranged from 99.5% to 107%, with an RSD of less than 1.2% for the three parallel measurements. These results demonstrated the high reliability and accuracy of the immunosensor in detecting IL-6 in real samples.

## 4. Conclusions

In summary, this study constructed an immunosensor for detecting IL-6 by integrating SNF with an immunorecognition interface on its outer surface and confining Co_3_O_4_ nanomaterials within its nanochannels. The SNF was grown on an indium tin oxide (ITO) electrode using the Stӧber solution growth method, and Co_3_O_4_ nanocatalysts were confined within the SNF channels via in situ electrodeposition. Co_3_O_4_ exhibits electrocatalytic oxidation capabilities toward luminol and hydrogen peroxide, enhancing the concentrations of luminol anion radicals and reactive oxygen species, thereby amplifying the ECL signal of luminol. In the presence of IL-6, the specific recognition between the capture antibody and IL-6 leads to the formation of immunocomplexes. This immunocomplex formation significantly decreases the ECL signal, demonstrating a signal “gating” effect triggered by the analyte and bio-specific recognition, which enables the sensitive detection of IL-6. The constructed immunosensor exhibits excellent selectivity and anti-interference capabilities, allowing for the detection of IL-6 in complex GCF samples. This immunosensing platform offers advantages such as simple fabrication and good detection performance, demonstrating potential for the detection of inflammatory factors in gingival crevicular fluid.

## Figures and Tables

**Table 1:** Determination of IL-6 using the fabricated immunosensor in gingival crevicular fluid.

Sample	Added (pg mL^−1^)	Found (pg mL^−1^)	RSD (%, n = 3)	Recovery (%)
Gingival crevicular fluid ^a^	0.100	0.107	0.7	107
	10.0	10.4	1.2	104
	1000	995	0.4	99.5

^a^ The gingival crevicular fluid was diluted by a factor of 100 using PBS (0.01 M, pH = 7.4).

## Data Availability

The data presented in this study are available on request from the corresponding author.

## Supplementary Materials

The following supporting information can be downloaded at: https://www.mdpi.com/article/10.3390/bios15010063/s1, Table S1: Comparison of IL-6 detection performance using different sensors.
