# Supplementary material for: Sensitive Detection of Biomarker in Gingival Crevicular Fluid Based on Enhanced Electrochemiluminescence by Nanochannel-Confined Co3O4 Nanocatalyst
Source: Biosensors (Basel). 2025 Jan 19;15(1):63. doi: 10.3390/bios15010063 (PMC11764429; doi:10.3390/bios15010063)
Supplement: Supplementary file 1 [file biosensors-15-00063-s001.zip › biosensors-3369731-supplementary.pdf]

Table S1 Comparison of IL-6 detection performance using different sensors.

**Detection Electrodes**	**Method**	**Probe**	**Detection Range ($pg\ mL^{-1}$)**	**LOD ($pg\ mL^{-1}$)**	**Ref.**
AuNPs/PPyNPs/SPGE	EC	Apt	$1\sim1.5\times10^{7}$	0.33	64
AuNPs/rGO/Au	EC	Ab	0.97~250	0.41	65
ErGO/AuPdNPs/HCPE	EC	Ab	$0.1\sim1\times10^{5}$	0.059	66
FC-PPN-Ab_2/IL-6/Ab_1/GO/GCE	EC	Ab	$2\sim2\times10^{4}$	1.0	67
rGO/Fe_3O_4/PDDA/CdSe/GCE	ECL	Ab	$2\sim2\times10^{4}$	0.65	68
BSA/Ab/GO/PANi/CdSe/GCE	ECL	Ab	$0.5\sim1\times10^{4}$	0.17	69
Ab_2-HRP/ACP/OAMs/IL-6/BSA /Ab_1/CTIL/$Ru(bpy)_3^{2+}$@AMCs/GCE	ECL	Ab	$1\times10^{-5}\sim9\times10^{4}$	3.5×10^{-6}	70
BSA/Ab/Co_3O_4@O-SNF/ITO	ECL	Ab	$1\times10^{-3}\sim1\times10^{4}$	6.4×10^{-4}	This work

AuNPs: gold nanoparticles; PPyNPs: polypyrrole; SPGE: screen-printed gold electrode; EC: electrochemistry; rGO: reduced graphene oxide; ErGO: electrochemically reduced of graphene oxide; AuPdNPs: gold palladium nanoparticles; HCPE: heated carbon paste electrode; FC: ferrocene; PPN: porous polyelectrolyte nanoparticles; GO: graphene oxide; GCE, glassy carbon electrode; PDDA: polydimethyl diallyl ammonium chloride; CdSe: cadmium selenide nanoparticles; ECL: electrochemical luminescence; PANi: polyaniline; HRP: horseradish peroxidase; ACP: acid phosphatase; OAMs: octahedral anatase mesocrystals; CTIL: carboxyl-terminated ionic liquid; $Ru(bpy)_3^{2+}$: Tris(2,2‘-bipyridyl)ruthenium(II) chloride, hexahydrate; AMCs: TiO_2 (anatase)mesocages.
